# Population Attributable Risk of Hyperuricemia in Hypertension Incidence in 20-74-Year-Old Population during a 10-Year Longitudinal Cohort: Yazd Healthy Heart Cohort

**DOI:** 10.18502/ijph.v49i10.4703

**Published:** 2020-10

**Authors:** Mojtaba MOHAMMADHOSEINI, Seyedeh Mahdieh NAMAYANDEH, Hosein FALLAHZADEH, Fatemeh MAJIDPOUR, Seyed Mahmoud SADR-BAFGHI, Mohammadhosein SOLTANI, Leila HADIANI, Mohammadtaghi SAREBANHASSANABADI, Hossein NOUGH, Ahmad KARIMI, Maryam ASKARI

**Affiliations:** 1. Research Center of Prevention and Epidemiology of Non-Communicable Disease, Department Biostatics & Epidemiology, Faculty of Health, Shahid Sadoughi University of Medical Sciences, Yazd, Iran; 2. Yazd Cardiovascular Research Center, Shahid Sadoughi University of Medical sciences, Yazd, Iran

**Keywords:** Population attributable risk percentage (PAR%), Hypertension, Hyperuricemia, Uric acid, Iran

## Abstract

**Background:**

The population attributable risk (PAR) percent has used widely in public health policy. We aimed to calculate the attribute risk of hypertension due to hyperuricemia by Levin’s formulas compare to direct PAR calculation method.

**Methods:**

This was a sub-study of Yazd Healthy Heart Cohort (YHHC). Overall, 1256 normotensive individuals were enrolled through multistage randomized cluster sampling and followed up for mean 9.8 years, from 2005–2015. The threshold cutoff point of the hyperuricemia was considered equal and more than 75^th^ percentile that equal to 5.5 mg/dl for men and 4.3mg/dl for women. To calculate the attributable risk of hyperuricemia in developing hypertension, two methods were applied. Levin’s formulas and direct PAR estimation by population risk calculation via exposure prevalence weighted formula. Multiple logistic regression was used for estimate of odds ratio (OR) of hyperuricemia in developing hypertension. We calculated Relative Risk (RR) from OR. The data were analyzed using SPSS software version 16. A significant level of 0.05 was considered.

**Results:**

Hypertension developed in 44.7% of individuals with uric acid level ≥ 75^th^ percentile vs. 35.6% of other individuals (*P*=0.024). Attributable risk (AR) of hyperuricemia in hypertension incidence was 9.1%. PAR of hyperuricemia for hypertension incidence by using two methods mentioned before was 6%, 5.8% respectively.

**Conclusion:**

The results of the study confirmed the noticeable contribution of hyperuricemia as an independent other risk factor for the occurrence of hypertension. PAR of hyperuricemia for hypertension incidence by using two methods almost near was 6%, 5.8% respectively.

## Introduction

Hypertension, as an important cause of morbidity and an almost controllable cardiovascular disease, is reported to be the fourth cause of premature death in developed countries and the seventh in developing countries (1,2). On the other hand experimental studies showed a causative role for uric acid in pathogenesis of hypertension (3,4). The concept of population attributable risk (PAR), frequently applied in public health programs, deals with the proportion of disease incidence prevented by eliminating the exposure factors in a target population(5). In other words, the PAR indicates the frequency of a disease attributable d to the risk factor in the total population. Furthermore, PAR can predict the effect of public health interventions on adverse outcomes (1). However, elimination of risk factor in population is not possible in real public healthy setting (6). Yazd Healthy Heart Cohort (YHHC) could show causal relationship with hypertension (7). On the one hand, the prevalence of HTN is growing in developing countries such as Iran (8, 9).

To quantify the impact of hyperuricemia in occurrence of hypertension in this article we reported PAR% by two known formula; Levin’s formula and direct calculation of PAR with exposure prevalence weighted estimation.

## Materials and Methods

This study was a historical cohort and a sub-study of YHHC. In the first phase, the enrolling of individuals into the cohort study was conducted from autumn 2004 to summer 2005. Overall, 2,000 people aged 20–40 yr in Yazd, Iran is being examined. Selection of individuals was done in a multi-stage random clustered manner. Length of the study period was 10 years and they were invited every year during the first five years and the last visit was after 10 years. In each visit, some lifestyle information such as dietary habits, physical activity and smoking, risk factors of cardiovascular disease including age, gender, hypertension, obesity and dyslipidemia, and some important biochemistry measures such as fasting blood glucose, cholesterol, triglyceride and uric acid were assessed and recorded Of total 2000 participants of YHHC 1265 participant without hypertension were enrolled in this study. Over-sell, 735 individuals with hypertension at baseline phase were excluded.

### Inclusion and Exclusion criteria

The criteria for entering the study include those residence in Yazd at least 1 year and the age in range of 20–74 yr, also not having hypertension at the beginning of the study.

### Ethical approval

The present study was ethically approved by the Shahid Sadoughi University of Medical Sciences’ Ethics Committee (ethics code: IR.SSU.MEDICINE.REC.1395.287). Informed consents were obtained from study participants at the initial and the follow-up phase. The present research is reported based on the strengthening the reporting of observational studies in epidemiology (STROBE) statement.

### Measurement of Blood pressure

Blood pressure was measured by a mercuric barometric device according to the standard protocol (10). After a minimum of 5 min rest, they were placed in a sitting position and then measured in right arm. The first and fifth Korotkoff sounds were taken as the systolic blood pressure (SBP) and diastolic blood pressure (DBP) respectively and the mean of these two measurements was recorded in each visit. Hypertension was defined as means all measured SBP at least 140 mmHg, or means two measured DBP at least 90 mmHg or the use of anti-hypertensive medication.

### Other risk factors

#### Uric Acid

Uris Acid was measured with biochemical auto-analyzer, model BT 3000 (Italy) and Man kits (Tehran, Iran). HU was defined greater than the 75^th^ percentile. The threshold cutoff point of the hyperuricemia 75^th^ percentile was equal to 5.5 mg/dl for men and 4.3mg/dl for women.

#### FBS

Fasting blood sugar (FBS) was after 10 hours fasting in serum by biochemical auto-analyzer, model BT 3000 (Italy) and Man kits (Tehran, Iran).

### Statistical analysis

The quantitative variables were reported as mean ± standard deviation and the qualitative variables were presented as frequency and relative frequency. To compare the differences between quantitative distributions of continuous variables, independent *t*-test was applied for the participants. In order to compare the frequency distribution of the qualitative variables, we run the chi-square test. Univariate analysis was also applied to examine the crude relationships between the HU and the risk of HTN. We used the multiple logistic regression models to assess the association between the HU and HTN after adjusting for the confounders of age, gender and FBS. We calculated PAR using the Levin’s formula and direct calculation of PAR with exposure prevalence weighted estimation. Levin’s PAR is calculated by the following formula (11).

(11.1) $$\%\mathrm{PopAR}=\frac{\mathrm{Pe}\times \left(\mathrm{RR}-1\right)}{\mathrm{Pe}\times \left(\mathrm{RR}-1\right)+1}$$

*Pe* =exposure prevalence in the target population, RR= relative risk of HU for HTN incidence

### Measurement of the relative risk (RR) using odds ratio (OR)

Relative risk was calculated by the following formula.

(11.2) $$\text{RR}=\frac{\mathrm{OR}}{1-\left[\left(q-\right)-\left(\mathrm{OR}\times q-\right)\right]}\;\;q-=\text{the}\;\text{incidence}\;\text{in}\;\text{the}\;\text{unexposed}$$

(11.3) $$\text{PAR}=\frac{\mathrm{Risk}\;of\;\mathrm{HTN}\;\mathrm{in}\;\mathrm{population}\;-\;\mathrm{Risk}\;\mathrm{of}\;\mathrm{HTN}\;\mathrm{in}\;\mathrm{Non}-\mathrm{HU}}{\mathrm{Risk}\;\mathrm{of}\;\mathrm{HTN}\;\mathrm{in}\;\mathrm{population}}$$

We calculated risk of HTN in population using prevalence weighted method:

(11.4) $$\begin{array}{l} \text{Risk}\;\text{of}\;\text{HTN}\;\text{in}\;\text{population}=\text{HTN}\;\mathrm{Risk}\;\mathrm{in}\;\mathrm{HU}\;\mathrm{patients}\times \\ \mathrm{prevalence}\;\left(\mathrm{HU}\right)\;\mathrm{in}\;\mathrm{population}+\text{HTN}\;\mathrm{Risk}\;\mathrm{in}\;\mathrm{non}-\\ \mathrm{HU}\;\mathrm{patients}\times \mathrm{prevalence}\left(\mathrm{non}\;\mathrm{HU}\right)\;\mathrm{in}\;\mathrm{population} \end{array}$$

HU is defined as the UA level of higher than 75th percentile, the prevalence of HU in the whole population was 23.8% according to base phase YHHC. The data were analyzed using SPSS (ver. 16, Chicago, IL, USA). A significant level of 0.05 was considered.

## Results

Table 1 presents the baseline characteristics of the patients. The mean age of the participants was 43.23 yr with a standard deviation of 14 at the baseline.

**Table 1:** The baseline characteristics of participants

***variable***	***HU***	***Non HU***	***Total***	***Sig***
Age (yr)	43.6 ± 15	43.09± 14	43.23 ± 14	0.55
FBS	94.5±27	96.89 ± 43	96.27 ± 39	0.35
Sex (male)	167(51.9%)	466(50.9%)	322(66%)	0.41
^*^P<0.05				

Table 1 shows individuals with HU and without HU were similar according to age (*P*=0.55, FBS (*P*=0.35) and sex too (*P*=0.41).

Table 2 shows that 44.7% of individuals with hyperuricemia and 35.6% of individuals without hyperuricemia developed hypertension. Individual with HU were more likely to have hypertension. Multiple backward stepwise regression analysis was conducted to examine the influence of HU on HTN (Table 3).

**Table 2:** Univariate analysis result of HTN risk and HU

***Risk factor***	***Normal, n (%)***	***Hypertensive, n (%)***	***Sig***
***HU 75^th^ percentile***			
Yes	110 (55.3)	89 (44.7)	0.024
No	410 (64.4)	227 (35.6)	
Total	520(62.2)	316(37.8)	

**Table 3:** Binary logistic regression of selected variables and the risk of HTN

***Risk factor***	***B***	***OR***	***CI***	***P-value***
Age	0.535	1.707	(1.503–1.939)	<0.001
Sex	0.127	1.136	(0.842–1.533)	0.405
Hyperuricemia 75^th^	0.395	1.484	(1.049–2.099)	0.026
FBS	0.006	1.006	(1.002–1.009)	0.004
^*^P<0.05				

The PAR percentage of HU was calculated as 6% according to the odds ratios obtained from the binary logistic regression models after adjusting for confounders. The PAR calculated for HU based on the exposure prevalence was also 5.8% (Table 4). The population attributable risk of HU in hypertension incidence was calculated as following.

**Table 4:** PAR of HTN caused by HU

***Levin’s population attributable risk***				***PAR weighted by exposure prevalence***	
HU 75^th^ percentile	^PAR, %^	RR	OR	PAR, %	Prevalence, %
	6%	1.27	1.484	5.8%	23.8%

Risk of Hypertension in whole population according to YHHC was 38%.

### PAR weighted by exposure prevalence

$$\begin{array}{l} \mathrm{risk}\;\mathrm{of}\;\mathrm{HTN}\;\mathrm{in}\;\mathrm{population}\\ \;\;\;=\left(44.7\%*23.8\%\right)\\ \;\;\;+\left(35.6\%*\%76.2\right)≅37.8\%\\ \mathrm{PAR}\;\mathrm{weighted}\;\mathrm{by}\;\mathrm{epousure}\;\mathrm{prevalence}=\frac{0.378-0.356}{0.378}\\ \;\;\;\;=5.8\% \end{array}$$

### Levin formula

$$\begin{array}{l} \mathrm{OR}=1.485,\;\;RR=\frac{1.484}{1-(\left(0.356\right)-\left(\left(1.484\right)*\left(0.356\right)\right)}\\ \%\mathrm{PopAR}=\frac{23.8\%\left(1.27-1\right)}{23.8\%\left(1.27-1\right)+1}=6\% \end{array}$$

## Discussion

This study examined causal association of HU in developing of hypertension and calculate PAR of HU in the risk developing of hypertension in 20–74 yr old in urban population of Yazd, a central city in Iran. The findings of the study confirmed a significant association between hyperuricemia and the development of hypertension. We calculated PAR of hyperuricemia with definition of 75^th^percentile and greater in the developing of Hypertension. We estimated PAR by two methods; Levin’s formula and the PAR estimation weighted by the exposure prevalence. PAR was estimated 6% and 5.8% respectively. On the other words, in the Yazd population 6% or 5.8% of hypertension incidence could be attributed to hyperuricemia. By eliminating hyperuricemia, the risk of developing hypertension in individuals with hyperuricemia decreased 5.8% or 6% over ten years.

PAR percent can be used for health policy aims so we can determine population interventions priorities. We usually do not have access to the incidence of disease to direct calculate PAR%. Practically we can use of other methods for estimating it such as Levin’s formula and PAR estimate using weighted population risk by exposure prevalence.

In both male and female patients, between hyperuricemia and developing risk of hypertension was significant relationship with OR 2.152 (95% confidence interval 1.324–3.498) and 2.133(95% confidence interval 1.409–3.229), respectively (12). This is same as our findings.

Systolic blood pressure increasingly was determined by diastolic blood pressure (B=1.09, 95% CI=0.99–1.19, *P*<0.0001), uric acid level (B=1.04, *P*=0.003), and waist circumference (B=0.131, *P*=0.033) (7). Findings of healthy United States adolescents’ study showed that increasing levels of serum uric acid are associated with high blood pressure (13).

After controlling biomarkers, eGFR, and total cholesterol, only uric acid and insulin independently predicted incident hypertension. The PAR of HTN regarding the top three quartiles of UA (i.e., uric acid ≥ 3.4 mg/dL) was 6.5 cases per 1000 women annually. The HTN estimated incidence rate was 14.6 cases per 1000 young women per year, the indicated that 30.8% of HTN incidence in young women was associated with a uric acid ≥ 3.4 mg/Dl (14). This study confirmed our findings.

For each 1-mg/dL increase in uric acid, the risk of developing hypertension increased 1.13 time in the adults (95% CI: 1.06 –1.20) (13). The pathophysiology role of sUA in hypertension and some underlying mechanisms are found but do not able to understand fully this relationship (15). UA lead to high BP and suggests a biologic mechanism that could lead to high BP in human (16). There was no association between uric acid and development of hypertension (4).

Elevated sUA was not an important risk factor for HTN. However, a recent study on the relationship between HU and risk of HTN introduced HU as an important risk factor in the development of HTN (12). Several explanations can be presented regarding the positive association between HU and risk of HTN. First, the elevated sUA level can reduce the circulating nitric oxide, activate the rennin–angiotensin system, increase the level of blood pressure, and therefore induce renal vasoconstriction. Second, the increased sUA may induce endothelial dysfunction; anti-proliferative effects caused by the elevated SUA impair the production of nitric oxide and the oxygen radicals generated by xanthine oxidase can cause HTN pathogenesis. Third, sUA enters the vascular smooth muscle cells and stimulates growth factors such as cycloxygenase-2 and platelet-derived. This in turn leads to smooth muscle cellular proliferation and secondary arteriolosclerosis (17).

The results of population-related contribution studies are limited. In addition, it is difficult to compare these results because the demographic groups are different and participants are various regarding their age, gender, and ethnicity. Therefore, we need to consider different risk factors in our measurements and set different cutting points for variables and their risk levels (18).

As the main strength of this study, we studied a large population selected from a prospective cohort. HU is significantly causal associated with the development of HTN.

To the best of our knowledge, this study was the first research that examined the PAR of HU in the developing risk of HTN in Yazd City. Our findings are not generalizable to the whole population of Iran. Lost data of uric acid level in baseline can be considered as our limitation.

## Conclusion

The findings of the study indicated the attributable contribution of HU to HTN among the studied population. In this regard, we can reduce the HTN incidence up to about 6 percent by lowering the UA level to less than the 75^th^ percentile. We recommend the authorities to take measures, conduct preventive interventions, and implement effective policies to reduce the burden of HTN caused by UH in Yazd City, Iran.

We can use the Levin’s formula and the PAR weighted by the exposure prevalence when we do not have access to disease incident and we need the use of its unbiased estimations for preventive intervention planning and in health policy priority determinations.
